# Moral Hypocrisy on the Basis of Construal Level: To Be a Utilitarian Personal Decision Maker or to Be a Moral Advisor?

**DOI:** 10.1371/journal.pone.0117540

**Published:** 2015-02-17

**Authors:** Wei Xiao, Qing Wu, Qun Yang, Liang Zhou, Yuan Jiang, Jiaxi Zhang, Danmin Miao, Jiaxi Peng

**Affiliations:** 1 Department of Psychology, Fourth Military Medical University, Xi’an, China; 2 Foreign Language Teaching and Researching Office of Basic Education Department, Chongqing Communication Institute, Chongqing, China; 3 Astronaut Scientific Research Training Center of China, Beijing, China; CSIC-Univ Miguel Hernandez, SPAIN

## Abstract

**Background:**

People encounter various moral issues that involve making decisions for others by giving advice.

**Objective:**

This study investigated the characteristics of providing suggestions for oneself versus providing suggestions for others in ethical decision-making and the differences between them based on Construal Level Theory (CLT).

**Methods:**

A total of 768 undergraduate students from three universities in China were randomly assigned to eight groups on the basis of a grid of two Construal Levels (self or others) by two different numbers of people saved (5 people or 15 people) by two problem situations (trolley problem vs. footbridge problem). The investigation examined participants’ decisions to opt to take action or refrain from action that would have the consequence of saving more people.

**Results:**

The main effects of Construal Level (F_1, 752_ = 6.46, p = .011), saving number (F_1, 752_ = 35.81, p < .001), and problem situation type (F_1, 752_ = 330.55, p < .001) were all significant. The interaction of the problem situation and saving number (F_1, 752_ = 1.01, p = .31), and social distance and saving number (F_1, 752_ = 0.85, p = .36), and interaction of the three independent factors (F_1, 752_ = 0.47, p = .49) were not significant. However, the interaction of social distance and problem situation (F_1, 752_ = 9.46, p = .002) was significant. Results indicated the participants utilized a component of utilitarian reasoning in the decision-making, and their behaviors appeared more utilitarian at low Construal Levels (CLs) compared to high.

**Conclusion:**

CLs, saving numbers, and problem situation significantly affected moral decision-making and exhibited significant interaction. Making decisions for oneself (low-construal) rather than giving advice to others (high-construal) was one important factor that determined whether the people were utilitarian or not. Utilitarian considerations are more relevant in impersonal dilemmas.

## Introduction

Chinese women badminton players were disqualified from the women’s doubles competition after being accused of “not using one’s best efforts to win” in the 2012 London Olympics. The coach and the players were doing everything to win the competition, but spectators criticized and accused them and the entire Chinese women’s badminton team for intentionally losing the game and for behaving contrary to the Olympic spirit. If you were the coach or one of the players who was facing either an outcome-based or a rule-based moral stance, which would you choose? If others ask your advice, what advice would you give them? This apparent conflict between decision-making for oneself and giving advice is one of the core areas of interest to morality researchers.

Each day, people encounter various moral issues that involve making decisions not only for themselves, but also for others by giving advice. In recent years, with the increasing prominence of Construal Level Theory (CLT), giving moral advice to others has become a keenly attentive area for investigation concerning ethical decision-making. Ethical decision-making is often characterized by two opposing modes of moral reasoning [1–4]. One is rule-based morality, in which a given action is right if it abides by relevant rules, or wrong if it does not. The other is outcome-based morality, in which an action is right if its consequences benefit people who are involved, even if rules are being violated. However, people often adopt different modes when deciding for themselves or giving advice to others; some researchers argued that this situation was called moral hypocrisy [3]. This study investigates the characteristics of making decision for oneself versus giving advice for others in ethical decision-making and views these differences through the lens of CLT.

This study also considers the concept of moral hypocrisy. Moral hypocrisy can be divided into two types. The first and more classic case is divergence of what people believe to be morally right, and how they actually behave [3]. The second and different type of moral hypocrisy is exhibited when one has double standards for morally evaluating one’s own actions versus similar actions performed by others [5]. Valdesolo and DeSteno defined moral hypocrisy in the second case as “a fundamental bias in moral judgment in which individuals evaluate a moral transgression enacted by themselves to be less objectionable than an identical transgression enacted by others” [6]. This current paper focuses on moral hypocrisy in the second case as a double standard in judging morality for oneself versus for others.

### 1.1 CLT and Social Distance

CLT states that people cognitively represent events at different levels of abstraction called Construal Levels (CL). The CL of an event is determined by the psychological distance between a subject and the event. For psychologically distant events, people tend to look for the essential general information, paying attention to the overall and abstract features, and focus on the outcome rather than the process (high CL). For psychologically close events, people tend to focus on peripheral and detailed features, and the specific procedure or process is priorities over outcome (low CL) [9].

CLT describes four types of psychological distance: Temporal; spatial (near vs. far); social (self vs. others); and hypothetical (events of a high probability vs. events of a low probability) [7]. These dimensions are formed relative to the zero point of the individual’s present personal experience. An event or activity can be categorized as high CL or low CL along each of these dimensions separately [8]. Social distance is the measure of how proximate a situation is to oneself and one’s own social group. For example, people exhibit closer social distance to themselves than to others, and they are closer to others in their social group than to those outside the group. Trope et al. suggests that social distance changes the responses of people to events by altering the way people mentally represent those events [9]. A greater social distance corresponds to a tendency to represent events in a few abstract features that convey the perceived essence of the events (high CL) rather than in more concrete details of the events (low CL). Informational and evaluative implications of low CL should therefore have a greater effect on responses about events affecting a respondent personally as opposed to events that affect others.

Differences in mental representations of events associated with large or small psychological distance can have significant evaluative consequences, i.e., a low CL should be more influential when psychological distance is small than when it is large, and vice versa Many studies discussed the influence and rules of psychological distance to options by studying desirability and feasibility [10, 11, 12]. Studies have found that differences in social distance can influence the desirability of alternatives in economic decision-making in daily life [13]. It seems that both the issue of outcome and feasibility places the focus on utilitarian reasoning, namely accomplishing a desired end. With deontic reasoning the focus is on the moral rightness or wrongness of specific features of the action. Thus, it is low CL oriented, but not primarily focused on feasibility. For example, in the trolley problem the feasibility of accomplishing the desired outcome is not in doubt, the problem is set up so that it is 100% feasible to switch the train to another track and save the people at the expense of killing fewer people. The moral issue, therefore, is not with feasibility versus outcome, it is with instrumental reasoning, i.e., feasibility and outcome versus non-instrumental reasoning, e.g., the intrinsic moral wrongness of intentionally causing innocents to die. Few studies have been conducted on the social distance of moral decision-making under the CLT framework. Researchers should give close attention to how social distance affects people’s mental representation and option preference in moral decision-making.

### 1.2 Moral Dilemmas

Empirical studies on moral decision-making have flourished in recent years. Morality is a fundamental theme of traditional social psychology and modern moral psychology, and plays an important role in everyday life [14]. Examining people’s responses to hypothetical moral decisions about life-or-death situations offers a good opportunity for cognitive psychologist to investigate the cognitive processes involved in moral decision-making. A moral dilemma is a problem situation in which an agent must decide between two or more discreet action choices that have explicitly delineated outcomes, which are morally significant. Among the many experimental paradigms, moral dilemmas have been the most common ones.

The moral dilemma paradigm is common for two reasons. First, an adequate moral dilemma design allows a methodology to systematically explore how distinct parameters modulate our moral judgment [15]. Second, it provides a good opportunity to induce moral conflict and investigate how people respond to these conflicts with basic moral intuitions. Consequently, a growing number of authors argue that moral dilemmas, such as the famous trolley problem dilemma, is a valuable means to study the factors that significantly affect the cognitive process of moral decision-making. [16–18]. Christensen and Gomila summarized the literature on moral dilemmas and pointed out that in spite of all criticisms, considering the complexities of moral decision-making can be influenced by a lot of factors, moral dilemmas provide a promising way to research these factors [19].

The classical experimental paradigms are the trolley problem, the footbridge problem, and related variants based on the theory of Haidt [20]. The moral decision-maker faces the same gains and losses, i.e., one dies to save five, in each version of the problem, but the means by which those outcomes are achieved differ. The following situation is given in Thomson’s version of the trolley dilemma: A runaway trolley is heading for five railway workers. The driver can change the course of the trolley by pulling a switch that will redirect the trolley to another track where it will kill only one railway worker instead of five. The footbridge problem describes a variation on the same dilemma, i.e., the participant, as the agent, had a choice between pushing with his own hands a large person onto the tracks to stop the trolley from killing five people. Research participants are asked to judge the morality of action versus inaction for each of these dilemmas. Results indicated that while most people intuitively accepted that the switch should be pulled in the first scenario, most people considered that they, the agent, should not push the large man, although the consequences were the same: One person is killed to save five [19]. Most participants faced with the trolley dilemma made a decision consistent with utilitarianism, in that they emphasized the maximization of overall lives saved, rather than the avoidance of causing death. However, most participants did not agree with the “one for five” option in the footbridge problem. Their judgment was not consistent with utilitarianism (deontic judgment) that emphasized moral rights and obligations. Greene et al. have argued that what differentiates the trolley problem from the footbridge problem is that the former is an impersonal dilemma, because the impact on the victims is executed by a switch without personal contact. However, the latter is personal because the impact on the victim is generated by participant’s muscles. However, an interesting finding was the existence of utilitarian in participants for moral judgment [16].

Christensen et al summarized that conceptual factors included personal force, benefit recipient, inevitability, and intention that have been shown to influence moral judgments [21]. They further pointed out that a utilitarian response would be easier to motivate the participant, as more people would be saved by such a response. Different numbers of “saved individuals” used in moral dilemmas allow researchers to explore whether participants are sensitive to utilitarian/consequentialist considerations or not. We assumed that there is no basis for distinguishing between these two cases in Normative Ethical Theory (NLT). Therefore, we should consider the differences in judgments about them to be based purely on moral hypocrisy and CL biases. Thus, this study further distinguishes “saving individual”, making moral decisions for themselves or for others, and the dilemma problems and explore all three to ascertain how they effect people’s moral decision making. It would be helpful to know how CL variables affect the moral decision making. Moreover, it might help NLT researchers to separate the morally relevant differences between the cases. Further, this study explores how the moral decisions that people make are influenced by CL.

## Method

### 2.1 Participants

The participants were 768 male undergraduate students from three universities in China. Their ages ranged from 18 to 24, with a mean of 21.54 (SD = 1.43). The participants were informed that they were participating in a psychological investigation in which no answer was considered right or wrong. They were also informed that they would be asked one out of eight questions and to make their decisions based on moral considerations alone. We distributed 768 inventories of which 752 were valid, thus obtaining a recovery rate of 97.92%.

All subjects were informed of the background, purposes, and significance of the research and provided their written consent before completing the study. The Ethics Committee of the Fourth Military Medical University specifically approved this study.

### 2.2 Materials

We adapted the research materials developed for a classic moral decision-making problem for the trolley problem and the footbridge problem. We made some adjustments to the original problems in order to manipulate the CL (social distance) and the number of people to be saved. A decision maker must decide whether he would take relevant actions or not, and provide answers with a rating from 1 (strongly disagree) to 6 (strongly agree).

The situation is given to the participants follows: A runaway trolley is barreling down the railway track. On the same track ahead, five/fifteen people are tied up and unable to move as the trolley heads straight for them. You/ Mr. Zhang are standing some distance off in the train yard, next to a lever. If you/Mr. Zhang pull this lever, the trolley will switch to a different track. You notice that one person is on the side track. You/ Mr. Zhang do not have the ability to operate the lever in a way that would cause the trolley to derail without losing a life e.g., holding the lever in an intermediate position for the trolley to go between the two sets of tracks or pulling the lever after the front wheels pass and switch again before the rear wheels pass. You have to rate from 1 (most likely to do nothing) to 6 (most likely to pull the lever) and show your should be done/your suggestion.

In another situation, a trolley is hurtling down a track toward five people. You/ Mr. Zhang are on a bridge and you/ Mr. Zhang can stop the trolley by dropping a heavy weight in front of it. A very fat man is next to you/him—your/his only way to stop the trolley is to push him over the bridge and onto the track, killing him to save the five/ fifteen people on the tracks.

### 2.3 Method and Procedure

We randomly assigned participants to one of eight groups on the basis of a grid of two CLs (self or others) × 2 saving numbers (5 people vs. 15 people) × 2 problem situations (trolley problem vs. footbridge problem) using a between-subject design. Responses to the decision-making problem were ranked by using a six-point Likert scale. Using a Likert scale measures the strength of the judgment made by the participants. A six-point scale was used, instead of the more common seven-point scale, in order to require participants to express a preference for one action over the other by denying them a mid-point choice. Saving number, CL, and problem situation were the three independent variables, while the response of the participants to the decision making problem were the dependent variable. Statistic analyses were conducted by using SPSS for Windows 16.0.

## Results

### 3.1 Main Effect

We tested the main effects of CL, saving number, and problem situation by using 2×2×2 ANOVA (means and number of participants in each cell are shown in Table 1). Results indicate that the main effects of all independent variables (CL, saving number, and problem situation, see Table 2) were significant. Compared with the footbridge problem, the mean in each cell showed that the participants demonstrated a higher tendency to sacrifice one for more people in the trolley dilemma (F_1, 752_ = 330.552, p < 0.001, r _effect size_ = 0.308). A higher saving number corresponds to more actions taken by the participants (F_1, 752_ = 35.813, p < 0.001, r _effect size_ = 0.046). Compared with participants giving advice to others, participants deciding for themselves were more likely to take actions (F_1, 752_ = 6.459, p = .011, r _effect size_ = 0.010).

**Table 1:** The means and standard deviation of willing to take action in different conditions.

situation	Saving number	self-decision			other-decision		
		N	M	SD	N	M	SD
Trolley problem	5 people	92	4.80	1.92	95	4.91	1.80
	15 people	93	5.48	1.32	90	5.52	1.17
Footbridge problem	5 people	94	2.62	1.94	95	2.09	1.65
	15 people	95	3.74	2.15	95	2.80	1.66
total	5 people	186	3.70	2.21	190	3.50	2.22
	15 people	188	4.60	1.99	188	4.10	2.16

**Table 2:** Effects of CL, number of people saved, and problem situations on willingness to take action.

Source		df	F	p	Effect size (Partial Eta Squared)
Main effect	CL	1	6.459	.011	0.010
	Number saved	1	35.813	<.001	0.046
	Problem situation	1	330.552	<.001	0.308
Interaction effects	CL × Number saved	1	0.853	.356	0.001
	CL ×Problem situation	1	9.463	.002	0.013
	Number saved × Problem situation	1	1.014	.314	0.001
	CL ×Problem situation× Problem situation	1	0.467	.494	0.001

### 3.2 Utilitarianism in Moral Decision-Making

**Fig 1:**
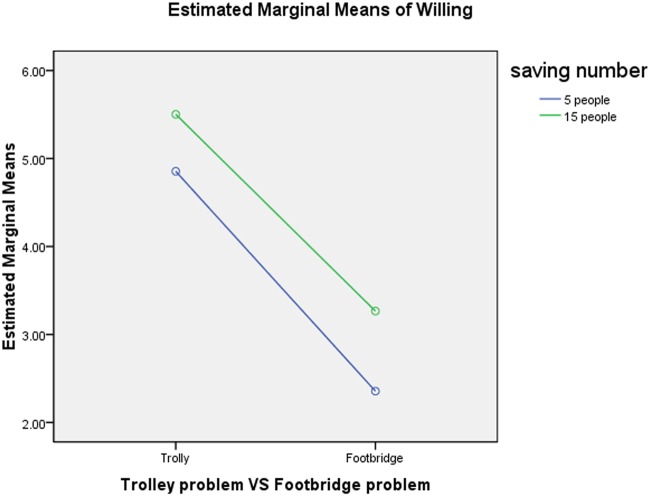
Interaction between moral situation and saving number. Note: Blue line, 5 people; Green line, 15 people; The interaction between Problem Situation and Saving Numbers showed non-significant (F1, 752 = 1.014, p = 0.31).

Problem situation and saving numbers (F_1, 752_ = 1.014, p = 0.31) exhibited non-significant interaction (see Fig. 1). A comparison between saving numbers in both the trolley problem and the footbridge problem indicate that participants were more willing to sacrifice one life for fifteen lives in either problem type. The greater willingness of participants to take action if more people will be saved indicates a tendency toward utilitarianism or at least a consequentialist component in overall moral judgment.

### 3.3 Difference in Moral-Decision Making and Advice Giving

**Fig 2:**
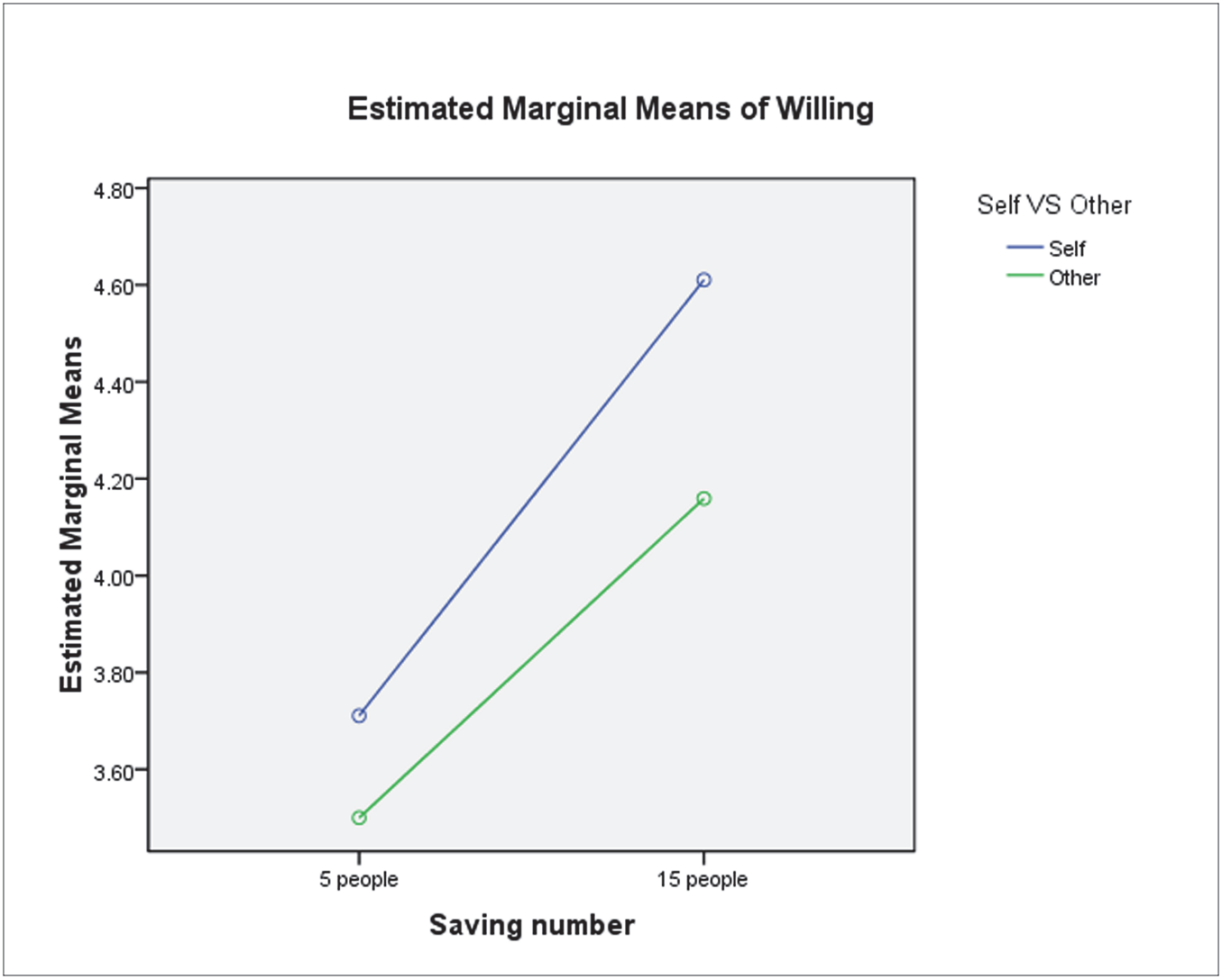
Interaction between moral situation and CL. Note: Blue line, decision for self; Green line, giving advice for other; The interaction between CLs and Saving Numbers showed non-significant (F1, 752 = 0.853, p = 0.356).

We further analyzed three-factor and two-factor interactions. CL and saving number (F_1, 752_ = 0.853, p = 0.356) exhibited non-significant interaction (see Fig. 2). Under low CLs, participants were more willing to take action to either save five lives or fifteen at the cost of one. This finding indicates that participants exhibited utilitarianism at low CLs and morality at high CLs.

**Fig 3:**
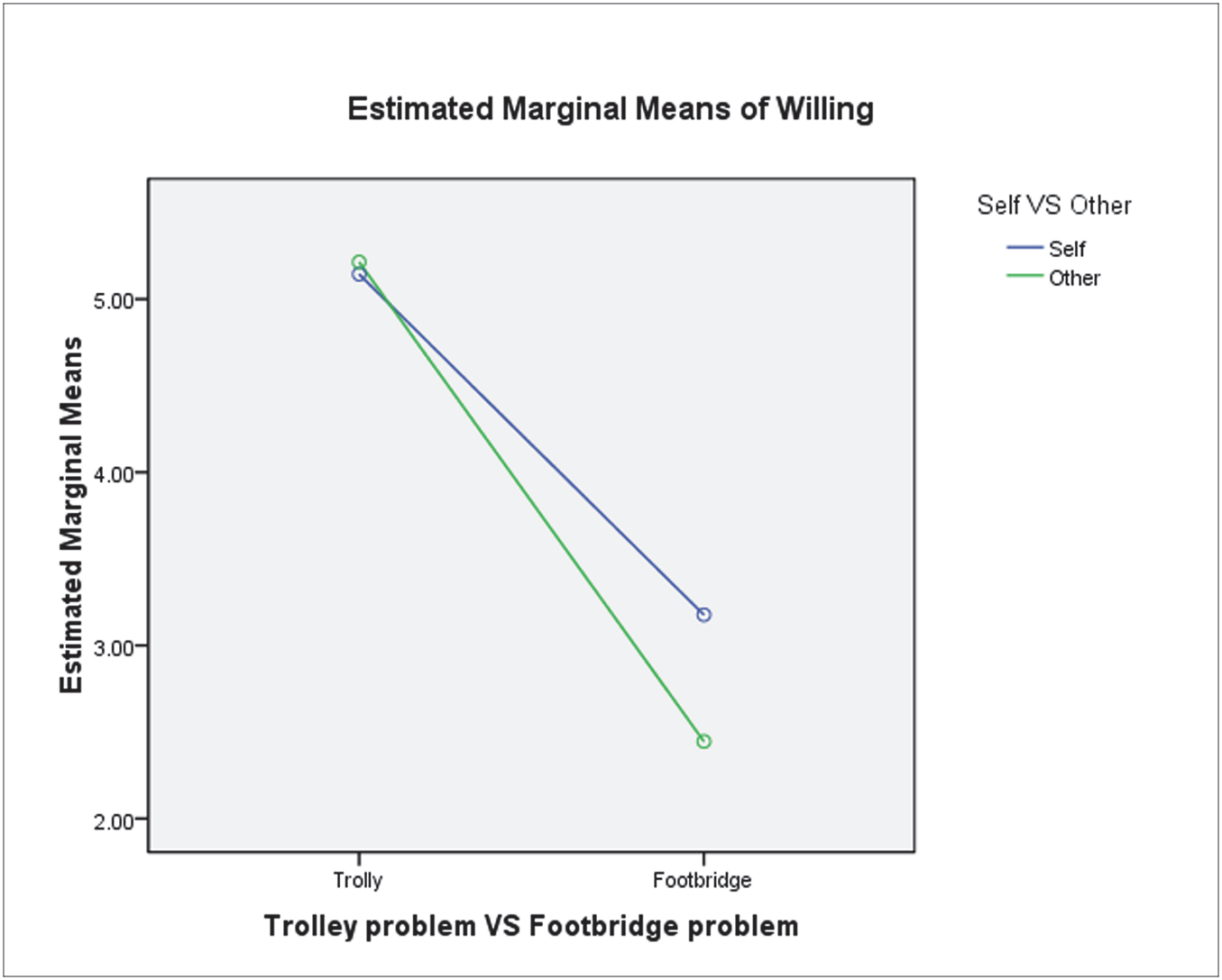
Interaction between saving number and CL. Note: Blue line, decision for self; Green line, giving advice for other; The interaction between CLs and Problem Situation showed significant (F1, 752 = 9.463, p = 0.021, r effect size = 0.013).

Furthermore, CL and problem situation (F_1, 752_ = 9.463, p = 0.021, r _effect size_ = 0.013) exhibited significant interaction (see Fig. 3). A simple effects analysis of the CL in each problem situation reveals that CL was non-significant in the trolley problem (F_1, 752_ = 0.125, p = 0.724). CLs did not measurably affect moral choices in the trolley problem. By contrast, it had significant effects in the footbridge problem (F_1, 752_ = 12.611, p < 0.0001). In other words, in the footbridge problem, the means showed that when making the decision for themselves, participants showed greater preference for taking action. Thus, in the footbridge problem, their judgments appeared more utilitarian at low CL.

According to this result, we could see that decision-making for oneself versus another was a key point that affected the attitude of the participants in making the morally dubious choice to push a person off the footbridge. When giving advice to others, people usually made the more conventionally morally acceptable choice. Self-decision or advice giving was the factor that determined whether the subjects of this research were utilitarian or not.

According to this result, we could see that whether decisions were made for oneself or for others was the key point that affected the attitude of the participants toward the footbridge problem. When giving advice to others, people tended to make the more conventionally moral choice not to push the person off the bridge.

**Fig 4:**
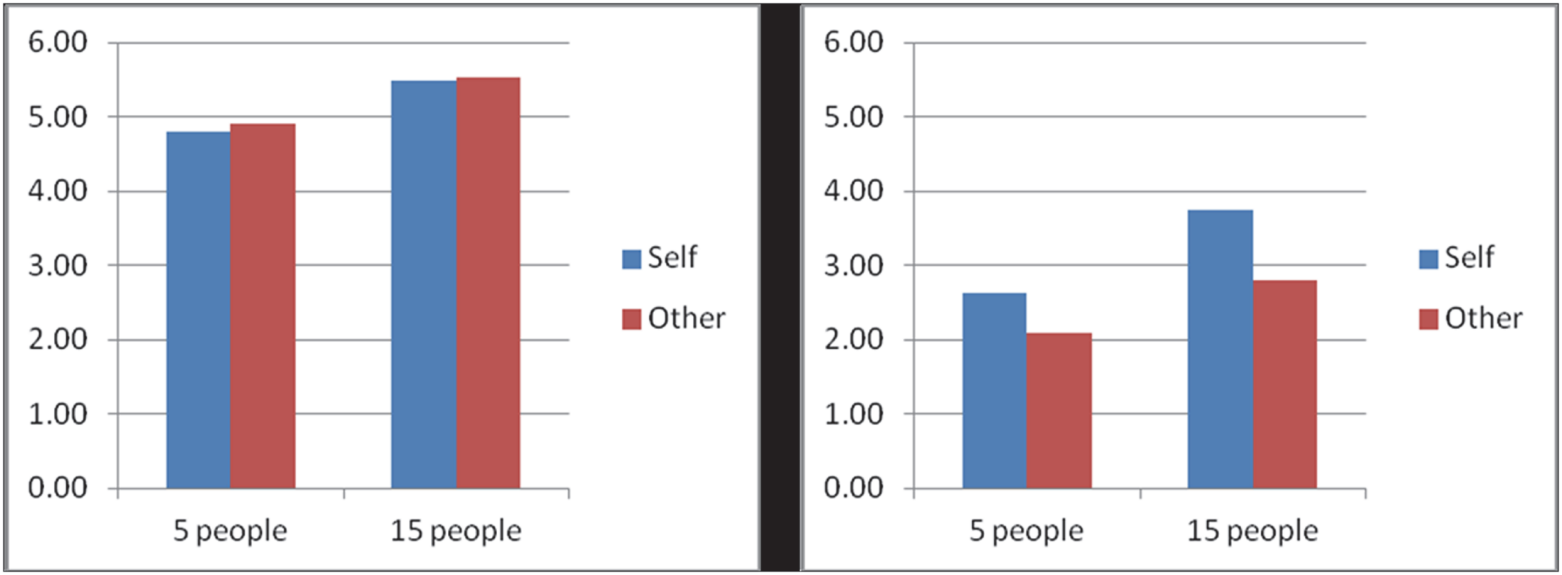
Interaction among saving number, CLs, and problem situation. Note: Y-axis, people’s willing to take action. The three-way interaction among CLs, Saving number and Problem situation showed non-significant (F1, 752 = 0.467, p = 0.494). However, people more likely to take action to save either one life for five lives or one life for fifteen lives in the condition of low CL.

CL, saving number, and problem situation (F_1, 752_ = 0.467, p = 0.494) did not show a significant three-way interaction (see Fig. 4). This result indicates that only in the footbridge problem, at low CL, participants were more willing to take action to save either one life for five lives or one life for fifteen lives. Compared with advice giving, making decisions for oneself indicated greater utilitarianism. However, CL had no significant effect on moral actions in the trolley problem.

## Discussion

### 4.1 Utilitarianism and Moral-Decision Making

The results of the study suggest that people are more willing to pull the lever if more people needed to be saved, and are also more likely to recommend this action to others. Moreover, saving numbers and the problem situation had non-significant interaction: Both in the trolley problem and footbridge problem, saving more lives had a greater impact in favor of taking action. This kind of moral decision-making exhibited cross-situational consistency. The results demonstrated that utilitarianism or consequentialist reasoning have at least some influence on people’s moral judgments [22–24]. However, the results also reveal that those judgments are not made exclusively on the basis of utilitarian reasoning. For if that were the case, then everybody would choose to take the action in both the trolley problem and the footbridge problem, since in every version of the problem more people are saved by taking action. Therefore, the results actually indicate that people use a mixture of consequentialist and deontic reasoning with some apparent cognitive biasing effects, too, as demonstrated by the effect of CL on the footbridge problem. This research shows that people are motivated, in part, by the desire to obtain the best outcome or choose the lesser of two evils.

Although high-quality culturally embedded and indigenous research is particularly important in advancing global research on moral decision-making, the majority of existing studies were conducted in Western cultures. Despite the fact that differences between cultures in the pattern and content of moral decision-making variables as well as in the causes and correlations of moral decision-making. This study provided acceptable evidence to claim that utilitarian reasoning plays a role in the moral judgments of members of more collectivist Asian cultures. This study broadened the empirical validity of previous studies that showed evidence of utilitarian reasoning in the moral judgments of the general public [16, 23, 25]. The study also contributed new evidence for expanding ethical theories that originated from Western individualist cultures to Asian cultures.

### 4.2 Social Distance and Moral Hypocrisy

This study also found that in the footbridge problem, when making moral decisions for themselves, people had a greater tendency to pull the lever, that is, to take the “one for five or fifteen” option. The interaction of social distance and saving numbers was not prominent, which suggests that differences between making decisions for themselves and making decisions for others had little or no effect on the impact of the number of people saved (five or fifteen). According to the expectations of CLT, when individuals represent themselves, they usually preferred low CL [7]. Therefore, when making decisions for themselves, they usually considered the moral choice and its results. With psychological distance among people, individuals tend to adopt a high CL when representing others. They seldom thought about results, but mainly cared about morality. Results of this study initially verified the above assumption and revealed the differences between making decisions for themselves versus giving advice to others. Compared with giving advice to others, people were more sensitive to self moral decision results and preferred to save more people: These findings could indicate moral hypocrisy.

Social psychology has always aimed at investigating the difference between the perception of oneself and that of others. CLT provided a systematic approach for solving this problem on the basis of cognitive representation. As mentioned in this paper, individuals had different concerns and representations for others and for themselves. The representations for others were more abstract, as they focus only on integrality and essential core attributes, whereas self-representations were more specific and gave more attention to periphery and detailed local features. Metaphorically, an individual who makes decisions for others is similar to a bird’s eye view over a forest and viewing an entire landscape, whereas a person who makes self-decisions is akin to close observation when looking at individual trees and leaves [23]. Kray and Gonzalez (1999) stated that only the superior dimension would be taken into consideration when giving advice [26]. While making decisions for oneself, individuals preferred equal weighting and valued both important and unimportant dimensions [27]. Therefore, different representations of self and others caused moral hypocrisy. Lammers et al found that an abstract focus increases hypocrisy because it increases flexibility in moral reasoning [3, 28]. As a result, “bending” how an action is construed is relatively easy. If a dilemma is perceived as “more of an abstract manner”, the “latitude of construal” is wider, and the alternative can be subjectively seen as more desirable [29–31]. The decreased flexibility constrains the “latitude of construal” and this increases the difficulty of how to choose and judge an action as more moral or not lie on making the decision for whom [25, 32]. This study confirmed the conflicting CL in the situation that was introduced at the beginning of this paper, in which athletes and audiences in the Olympic Games had to make decisions. The conflicting CL between themselves and others caused different decision results, which induced moral hypocrisy.

### 4.3 Utilitarian Considerations Are More Relevant in Impersonal Dilemmas

Another finding was that the effect of social distance on people’s decision-making was restricted by the situation of the dilemmas, with utilitarian considerations being more relevant in impersonal dilemmas. This effect was observed because of the significant main effect of social distance and the interaction between social distance and the problem situation, but not of three factors. Whether the number of people to be saved was five or fifteen, social distance had less effect in personal dilemmas, and self-decision behavior or advice for others was consistent. In the footbridge problem, or impersonal situation, the number of individuals and social distance had a profound effect. Self-decision and advising others were significantly different, which indicates moral hypocrisy. In general, utilitarian considerations have more weight in impersonal dilemmas. Therefore, this study also provided new evidence for exploring the weight of utilitarianism in moral decision-making. Utilitarianism contends that consequences are the only reason for selecting the morally best option. However, Foot (1967) stated that according to utilitarianism [33], it can be a morally right action for a surgeon to transplant one person’s organs to five others for the reason of saving more people; this is an option that a strong majority rejects. In general, utilitarianism misses the morally relevant difference in different situations and the weight of non-utilitarian considerations. This study provided the most direct evidence of this difference. Another critique states that utilitarianism requires applying the same moral weight to the interests of self and others [34]: Our study did not support this view. To recall, the trolley problem dilemma is impersonal, while the footbridge problem is a personal one [16,35]. This study empirically suggested the different moral weight of utilitarian considerations in personal or impersonal situations. Utilitarianism fails to capture the difference between whether a situation is impersonal or not. More importantly, it does not recognize the weight of moral considerations in different situations.

In other words, utilitarian considerations are situational according to different moral dilemmas. Hypocrisy is different from immorality. Hypocrites generally have a clear knowledge and understanding on what is right or wrong because hypocrites will not accept moral violations such as spitting on the ground or smoking in public [20]. Nonetheless, similar to many other forms of motivated reasoning, as CLT is different, hypocrisy is ultimately a form of cognitive flexibility [16, 36, 37]. Thus, hypocrites assign different moral weights in judging for themselves than for others, while normal morality requires that we assign moral weights impartially across self and others. In the face of huge personal interests, cognition processes have enough flexibility to acquiesce to demands like those of coaches to athletes. And as we described in the introduction, they try every means to win when they are involved. However, when they become the audience, they require players to obey the rules of the game strictly. Utilitarian considerations are not absolute but limited by certain situational factors or the weight of moral. The situation can be likened to throwing a sprat to catch a herring: Athletes make utilitarian moral decisions and sacrifice their own opportunities to help teammates win. Self-decision and giving advice to others would become highly consistent because players would not break the moral bottom-line and act in an unfair manner, which would make the other players unable to play.

Our study has a number of limitations. Its major shortcoming is that it adopted the moral dilemma paradigm. Thus, this study has many problems that are observed in morality studies. For example, decisions made in reality are not consistent with those that could be triggered by different dilemmas. In addition, a between-subject design involves confounding factors, such as personalities, families, and financial situations. Finally, this study attempted to investigate the cognitive mechanism of moral hypocrisy on the basis of CL, but the current study only connects to CL through inference, based on the assumptions of CLT, i.e., increasing social distance corresponds to higher CL. Future studies may explore more substantive factors in the reasoning process and mechanism.
